# Experimental Challenge Models and In Vitro Models to Investigate Efficacy of Treatments and Vaccines against Amoebic Gill Disease

**DOI:** 10.3390/microorganisms9040710

**Published:** 2021-03-30

**Authors:** Jemma Hudson, Barbara F. Nowak

**Affiliations:** Institute for Marine and Antarctic Studies, University of Tasmania, Launceston 7250, Australia; jhudson6@utas.edu.au

**Keywords:** Amoebic Gill Disease, experimental infection, in vivo, in vitro, efficacy, *Neoparamoeba perurans*, vaccine testing, treatment testing

## Abstract

Amoebic Gill Disease (AGD) severely affects salmonid mariculture due to fish losses and costs associated with management of the disease. Continued research into management solutions, including new treatments and vaccine development, is highly important for the future of salmonid production worldwide. This requires both in vitro (both pathogen only and host-pathogen models) and in vivo (disease challenge) testing. Challenge models are still widely varied, in particular with regard to: infection methods (cohabitation or immersion), source of the pathogen (isolated from infected fish or cultured), infectious dose, environmental conditions (in particular temperature) and the endpoints across experimental treatment and vaccine studies which makes comparisons between studies difficult. This review summarises in vitro assays, the challenge methods and endpoints used in studies of experimental treatments and vaccines for AGD.

## 1. Introduction

Amoebic Gill Disease (AGD) is a potentially fatal disease that primarily affects salmonids farmed in marine environment, although it has been reported from other farmed marine species [1,2,3,4,5]. First described in 1986 [1], the disease has become a significant expense to Atlantic salmon production globally, with production costs in Tasmania increasing by up to 20% due to mortality, costs of treatment and losses in growth associated with the disease [4]. The disease can cause mortalities of Atlantic salmon in Tasmania of up to 10% per week once established on a farm [6].

Atlantic salmon affected by AGD generally become lethargic and experience respiratory distress often resulting in suffocation and mortality [7,8]. The gills of affected fish become hypertrophic, with hyperplastic epithelium resulting in fusion of the gill lamellae and sometimes filaments, which can be visible in gross pathology [7]. Interlamellar vesicles can form between the affected gill lamellae, and excess mucus can be seen, as a result of an increased activity in mucus cells and increased numbers of mucus cells [9]. The lesions are most frequent in the epithelium in the dorsal part of the gill [9].

*Neoparamoeba perurans*, the aetiological agent of AGD, is perhaps the most important amoeba in salmonid aquaculture, however little is known about its biology. The amoebae were first described and shown to cause AGD in 2007 and until this point the causative agent had been thought to be *Neoparamoeba pemaquidensis* [10]. This is in part due to the nature of the amoebae from genus *Neoparamoeba*, which cannot be distinguished by their morphology alone [11,12]. Amoebae from this genus contain a nucleus-associated eukaryotic endosymbiont which can be observed as an additional nucleus in histological sections and smears [13,14]. The shape of the amoebae can be variable, from round when in stressful conditions to irregular with pseudopodia [11,12].

Being a marine species, *N. perurans* is vulnerable to low salinities. This means that high salinity is a risk factor for AGD outbreaks, however other risk factors are not as clear, with outbreaks occurring at a range of temperatures globally [1,15,16]. In Tasmania, outbreaks have occurred at temperatures as low as 10 °C [17]. It is thought that rather than the amoebae being more common at certain temperatures, infections are more likely to occur at temperatures that are abnormally high for the host species in their region [4]. Other environmental factors that affect occurrence of AGD are cage fouling, low water exchange, and stocking densities of fish. More cases of AGD have been seen in fish in heavily fouled cages, and with low water exchanges [18]. Cases of AGD were more severe with higher densities of fish in a laboratory experiment [19] and based on the outcomes of an industry questionnaire [18]. *N. perurans* is capable of surviving on fish post-mortem, and successfully multiplying and infecting naïve fish, which makes the effective treatment and management of affected animals even more vital [20].

The most used treatment for AGD is bathing the affected fish in freshwater. Generally, this involves a 2–4 h bath, however shorter-term bathing has also been successful in reducing AGD [21]. Water hardness has been shown to have an effect of freshwater bath efficacy—the use of softened water significantly reduced visible amoebae on gills from 73.9% to 40.9% and increased time to next bath [22]. Hydrogen peroxide baths can successfully treat AGD, however the toxic nature of this treatment makes it less ideal and potentially harmful, particularly above 13.5 °C or for fish that are severely infected [23]. Experimental Chloramine T treatment significantly reduced amoeba density with the same effectiveness as freshwater [24]. There is a need for development of new effective treatment which requires in vitro and in vivo testing.

Experimental vaccines have been trialed with little success so far, despite evidence that salmonids can develop some resistance to the disease [25]. Attempts to increase resistance using immunostimulants have been successful and immune responses give indications that vaccines could be developed [26,27]. The injection vaccines that have been tested include the use of paramoeba antigens and a recombinant protein, neither of which have influenced infection rates of salmonids [28,29]. An immersion vaccination using amoebae antigens from both cultured and wild type strains of *N. perurans* showed no increase in survival of the vaccinated fish [30].

Specific guidelines have been developed for vaccine trials in fish, to ensure clinically relevant data are collected without unnecessary harm to subject animals. These guidelines, created in 1981 [31], require that a minimum of 25 fish per replicate are used, with control and treatment groups being at least duplicated. During the challenge stage of these trials, a minimum of 60% of control fish must be infected with <20% variation between replicates of all groups [31]. This ensures the results are reasonably consistent between replicates. The fish used in experiments must reflect the animals that would be vaccinated in commercial settings in size, age, and life stage—and if possible, come from the same stock as commercial fish. Further guidelines by the European Medicines Agency recommend fish be monitored for a minimum of 14 days with daily checks for mortality [32].

The current methods used to manage AGD are not ideal: freshwater bathing is costly and is becoming less effective with number of baths increasing with time, therefore research is continuing to explore alternate methods of treating or vaccinating fish to prevent AGD. The methods used to infect animals in studies of vaccines and treatments are important to understand how effective and accurate the experimental trials are. The severity of infection of fish in these studies is a crucial factor in understanding the success of experimental treatments, so ensuring that initial infection rates are understood is important.

The endpoint of the challenge is important to consider for both accuracy and consistency of results across studies as well as for the ethical treatment of study animals. The success of treatments or vaccines can be measured through the survival/mortality of fish over the length of a study, severity of gill lesions, or amoebae load on the gills.

This review discusses both in vitro and in vivo testing methods, including challenge methods and the endpoints for vaccine and treatment testing used in studies of AGD.

## 2. In Vitro Testing

In vitro assays are often used for screening experimental treatments against cultured pathogens. In vitro testing has been used to assess potential treatments against AGD [23,33,34]. Usually, those tests involve exposing *N. perurans* (either cultured or isolated from the gills of infected fish) to different concentrations of the treatment. The efficacy is measured by quantifying either dead or alive amoebae after experimental exposure to different concentrations of the chemical compound. The main methods to quantify dead or live amoebae are either Trypan blue exclusion test or Neutral Red inclusion test. For example, efficacy of hydrogen peroxide and Chloramine-T as amoebicidals were tested using trypan blue exclusion test [33], and effective concentrations of hydrogen peroxide were determined using Neutral Red inclusion assay [23]. Trypan blue exclusion test only indicates the retention of membrane integrity and not necessary viability; therefore Neutral Red is preferred as the dye is actively taken up and transported into vacuoles and vesicles, indicating cellular functions. More recently high throughput tests bioassays based on metabolic energy production and cellular membrane integrity to distinguish between amoebistatic and amoebicidal actions were shown to be highly efficient for screening of potential treatments against on *N. perurans* [34].

However, exposing the pathogen only to an experimental treatment ignores any effects of host-pathogen interactions, with some reducing the exposure others potentially intensifying its effects. Therefore, in vitro models which include host factors have been developed. To visualize effects of paramoebae on fish cells, models which allowed observation of cytopathic effect of the amoebae were developed with RTgill-W1 and *N. pemaquidensis* [35] or CHSE-214 cells and *N. perurans* [36]. Isolated perfused gill model was developed to study Atlantic salmon gill conditions, including AGD and was applied to understand AGD pathophysiology and could be used to evaluate potential treatments, but still requires killing fish to isolate gill arches [37]. Another in vitro model, utilizing RTgill-W1 seeded onto Transwell^®^ inserts, maintained asymmetrically with apical sea water and exposed to *N. perurans*, showed similar host responses to those from in vivo experiments and cytopathic effect by 72 h, suggesting potential for treatment testing [38].

## 3. In Vivo Challenge Methods

Cohabitation of infected and naïve fish represents the challenge method best replicating natural infections. Infected fish can be placed in experimental tanks with naïve fish, or naïve fish can be placed with infected fish in a separate tank before being transferred to their respective experimental tanks [39,40,41]. The earliest study using this technique for AGD used a ratio of 15 infected to 40 naïve fish (1:2.07), which caused AGD infection after 7 days with all fish infected by day 24 [42]. Another study using the same ratio of infected and naïve fish across three species showed infection by day 3 [39]. Two other studies used a lower ratio of 1:3 infected to naïve fish and infection occurred within 21 and 3 days, respectively [28,43]. This variation between studies using similar infection methods highlights the difficulty in controlling infection rates when this challenge method is used.

The more common method of challenging naïve fish is through direct exposure to *N. perurans*, although this technique changed over time, with the development of improved isolations from gills of infected fish and in more recent cases culture of the amoebae. Wild amoebae can be sampled directly from gills of infected fish [44]. In early studies, gill mucus from infected fish was centrifuged with seawater to isolate the amoebae and placed into experimental tanks [44]. The inoculations of amoebae were partially purified by allowing amoebae to adhere to Petri dishes after being isolated [45]. These changes in methods allowed the minimum infective dose to drop from 230 cells L^−^^1^ to 10 cells L^−^^1^, however in reality, the doses used in experimental trials vary widely (Table 1). The method of presenting fish with the inoculant can vary: the inoculant can be added directly to the tank, or to a smaller tank used only for the infection (usually a few hours).

The concentrations of *N. perurans* to which fish are exposed during the direct exposure challenge are very high in comparison to those measured in water on affected farms [60,61,62]. The need to use more realistic concentrations of *N. perurans* to ensure that overexposure to the pathogen does not adversely affect potential treatments or vaccines should be considered [63]. Despite the minimum effective concentration of *N. perurans* in challenge studies being as low as 0.1 amoeba L^−^^1^ [64] and the concentrations measured on fish farms usually around 1 amoeba L^−^^1^ [60,61,62], the range of concentrations used in experimental trials is much greater, even as high to 5000–10,000 amoeba L^−^^1^ for some studies (Table 1). The severity of AGD lesions in Atlantic salmon is correlated with the exposure concentration and the time to lesion development is shorter with higher exposure concentrations [44], so it is likely that higher concentrations are used to ensure that the desired severity of AGD can occur within shorter time [45,65]. Onset of AGD lesions can be observed 2–4 days post infection for concentrations higher than 1000 cells L^−^^1^ and more than a week in lower concentrations (Table 1). There are some exceptions however, with one study that used only 100 cells L^−^^1^ resulting in onset of AGD lesions after only 4 days [53]. The most commonly used concentrations for exposure in the experimental tanks are 100 and 500 cells L^−^^1^ (Table 1), the latter being based on the highest concentration used during the development of the standard adherence method and causes lesions in 30% of gill filaments in 14 days [45]. However, it is important to ensure that the challenge protocol is realistic and reflects field exposure, so excessive challenge pressures do not result in rejection of a treatment or vaccine which could be efficient in the field.

Often for a challenge by direct exposure to wild *N. perurans*, the amoebae need to be collected in batches to accumulate enough for the required concentrations across all replicates. Batches of amoebae can have different levels of virulence, resulting in varied severity of gill lesions and therefore have a significant effect on the results of a study if experimental groups are exposed to different batches [19]. To reduce the potential variability across groups, batches should be equally divided between tanks until the desired concentration is reached [19]. If cultured amoebae are used it is important to test their virulence as it has been shown to decline with culture time [36]. After three years, one clonal culture of *N. perurans* became anti-virulent, with a general trend for lower virulence in cultures that were maintained for longer periods [36]. Differences in virulence as well as differences in growth dynamics were seen in different clonal cultures of *N. perurans* [65,66]. Significant differences in gill scores were seen between different cultures using the same challenge concentrations of 500 and 5000 cells L^−^^1^ [65]. It is important to note that both clonal and polyclonal cultures have been used for challenges and so far there is no axenic culture for *N. perurans* so there is a potential for involvement of bacteria and not just the amoebae [65,66]. Although clone cryopreservation shows some promise, no 100% reliable method has been developed yet [67].

The tank system used for a challenge can affect the required concentration of amoebae. The use of recirculating aquaculture systems for experimental trials is convenient and allows for consistent water quality, however the aspects of these systems that allow for clean, high quality water, act negatively during challenge periods as they can affect the rates of infection. Recirculating pumps can move water across biofilters and temperature control units—causing amoebae to be transported away from tanks resulting in concentrations to differ between replicates [19]. *N. perurans* numbers in duplicate samples of water were highly variable during an experimental challenge [63], suggesting uneven distribution of the amoebae in aquatic environment, which may result in significant differences between replicate tanks [64].

Nominal concentration based on counting the amoebae and adding them to a known volume of water is most commonly reported in the challenge trials while measured concentration is not very common despite qPCR methods available for this purpose. However, immersion methods (exposure to pathogen in water) are known to result in uneven exposure of individual fish [64]. Intra-peritoneal and intra-muscular injection of pathogens are used for other diseases and allow uniformity of challenge doses across all fish in a study [68]. No such method of infecting individual fish directly has been developed for AGD challenge studies, as *N. perurans* is an ectoparasite and injection would not be a relevant method of infection. The methods of cohabitation and direct exposure to amoebae do have the benefit of more accurately imitating the exposure fish would experience in the wild. Instead, the difference in the concentration added versus the concentration reaching naïve fish can be reduced by turning off recirculation pumps and protein skimmers for 4–6 h after the addition of amoebae as recommended by Crosbie et al. (2010) [19]. Even in static systems concentrations of amoebae added to the surface of tanks are distributed throughout the water column without aid due to movement of fish [63].

Between the two methods most used in challenges, direct exposure of the fish to amoebae allows for more control over infection levels. Variability in virulence between batches or clones has been reported in both wild and cultured *N. perurans*. However, this can be overcome through spreading batches or cultures evenly across experimental tanks. The use of cultured *N. perurans* for challenges has the potential to cease the need to maintain infection tanks used for isolations of wild *N. perurans*, thereby reducing the number of fish needed to continue studies of treatments and vaccines of AGD. However, the potential of cultured *N. perurans* to lose virulence over time means that new cultures would need to be created regularly. Cohabitation methods vary in exposure to infectious load if fish are challenged in different tanks in a study, and exposure is difficult to reproduce between studies. Cohabitation does however, replicate the environment in which farmed fish are exposed to AGD more accurately than direct exposure.

Other factors which should be taken into account when comparing results of two different challenges is Atlantic salmon origin and characteristics and water quality. Fish origin, size, age, health status should be reported. Atlantic salmon farmed at different geographical locations differ, for example geographical origin of Atlantic salmon affects mucus characteristics [69] which are important in AGD [70]. AGD resistant families are increasingly available and while they have to be tested as the commercial target for a vaccine or treatment, they may require higher concentrations of the amoebae, longer challenge times or different environmental conditions. Water quality, in particular temperature can affect the challenge outcomes and vary between trials (Table 1), at least partly reflecting environmental conditions on salmon farms in the geographic area of interest.

## 4. Endpoints of In Vivo Challenge Studies

The endpoint of the challenge experiments depends on the purpose of the study, and the treatment or vaccine being tested. Certain standards exist to evaluate the success of vaccines for example, the relative percent survival (RPS) of experimental animals (% vaccinated mortality/% control mortality). While testing novel vaccines, mortality of control groups should be greater than 60% (with mortality unrelated to the relevant pathogen being less than 10%) to ensure survival of vaccinated groups is related to the presence of a vaccine and not due to chance [31]. Survival and mortality are used as measures in studies of treatments, with a mortality level of 70% frequently being used as an endpoint (Table 1). The use of mortality as an endpoint in experimental studies has implications for ethics as it ultimately relies on studies being designed around the death of a certain number of animals. As a result, recommendations to use the smallest number of animals to achieve a statistically significant result have been given to reduce potential suffering [32] or to use morbidity instead of mortality. Often however, vaccine studies do not reach the optimal RPS (Table 1). A lower RPS may be necessary if the concentration of amoebae used to challenge fish is not high enough or the challenge strain not virulent enough to cause high mortality [71].

Alternately, comparative gill pathology can be used to measure the severity of infection at the end of the experiment. As the number and severity of gill lesions is reflective of the severity of AGD, levels of infection can be measured by gross pathology. Gross gill score involves examining the whole gill of anaesthetised live, or euthanised fish and giving a score from 0 to 5 that reflects the level of infection based on number and size of lesions, necrotic streaking and mucus patches [72]. The lightest level of infection (1) is indicated by one lesion, while the heaviest level of infection (5) is indicated by lesions covering more than 50% of the gill filaments [72]. This method is relatively non-invasive and often used in commercial settings to measure levels of infection in farmed fish, so it is highly relevant for the salmon industry. However, measurements using this method must be interpreted with caution, as lesions can result from pathogens other than *N. perurans* which can potentially lead to overestimation [53]. The appearance of lesions on gills can be affected by prolonged fixation resulting in gross gill scores not reflecting the quantitative assessment of the infection severity [55]. Finally, assessment of gross lesions can be affected by the observer’s experience and level of lighting used.

Histological sections of the gills can be used for counts of filaments affected by AGD lesions or counts of lesions/filament or size of individual lesions. Histology estimates are more invasive as they require fish to be killed, however, they are more accurate, and can include amoebae load on the gills which cannot be measured in gross lesion counts. The results are usually presented for one gill arch (standardized—often second left) as the percentage of gill filaments affected in one section. Sometimes size of AGD lesions is determined by counting interlamellar units involved in each lesion. Gill histology is generally taken from a section of a single gill arch, while gross lesion counts are taken from all gill arches in an individual [9,19]. Estimates taken from gill counts and gill histology have been shown to indicate similar levels of AGD, although gross lesion counts were higher for some individuals [19]. Amoebae load from gill histology is estimated by manually counting individual amoebae using a compound microscope and are limited to the sample processed for histology and usually done only for one histological section.

Another method for measuring amoeba load on the gills of the fish is qPCR analysis of DNA samples taken from gill swabs or gill tissue. However, this method should be used together with gross score or histology as it does not assess presence or severity of the lesions, but only the presence of the DNA of the pathogen. Gill filaments can be removed from euthanised fish, placed in ethanol or RNAlater and stored for later testing [21,55]. Mucus swabs taken from gills of infected fish can be used in PCR testing and provide a less invasive method of doing amoebae counts. A range of PCR methods have been developed, which are specific to the *N. perurans* 18S rRNA gene and the results can be quantified as 18S rRNA copy number [3,36,60,61,73,74]. The area of the gill that swab samples are taken from differs across studies, however the third and fourth gill arches tend to contain the highest load of amoebae [41]. Using mucus swabs for detection and quantification of amoebae tends to be more variable than using samples of gill filaments. In the PCR assay from swabs taken during biopsy were only amplified in one out of six samples, while those from swabs taken during necropsy were amplified in five of the six samples, despite DNA from gill biopsy samples being amplified five of six times [3]. Results of PCR tests from mucus swab samples can vary depending on the material of the swab used—Calgiswab swabs and cotton swabs are more absorbent and so less of the sample is recovered during agitation [41].

A combination of gross pathology, histological and molecular methods is frequently used as endpoints in challenge studies as multiple samples can generally be taken from the same individuals (Table 1). If severity of the disease is to be determined presence of lesions and the pathogen need to be confirmed. The endpoints used can affect challenge design and interpretation of results. The relevance of the endpoints to the end-user should always be considered.

## 5. Conclusions

As management solutions, either vaccines or treatments, are generally developed for aquaculture industries, it follows that the challenge models used to develop these vaccines and treatments should be as close as possible to the natural transmission methods so they are the most relevant to industries. Due to the nature of AGD differing in its epidemiology globally, it would be reasonable for experimental studies to develop management solutions that apply to local aquaculture industries.

If that were to be the case for AGD challenge models, cohabitation would be the infection method used, resulting in naïve fish being exposed to low concentrations of amoebae. This model is more relevant to salmonid industries, which are the reason that most studies are being conducted. However, this would result in studies having little reproducibility, which could reduce the efficiency of finding a reliable treatment or vaccine.

Experimental temperature should be considered. For experimental challenges to be reproducible, the same temperature would have to be used across all studies, whereas to simulate natural conditions during experimental trials, temperatures that are associated with AGD in the region where the study fish were sourced should be used. As the range of temperatures at which AGD is developed in aquaculture systems varies globally depending on the tolerance of the host species, it is generally the case that temperature varies depending on the study region, reducing replicability between trials.

For highest relevance to the salmonid industry, the use of cohabitation as a challenge method should be recommended, alongside region relevant temperatures and low infection rates. Although this would reduce the reproducibility of studies, many current studies cannot be compared anyway due to differences in temperature or challenge concentration or source of the amoebae or salmon. Alternatively, realistic concentrations of amoebae should be used in challenge models. The most relevant endpoints to aquaculture industry should be used, for example time to reinfection or gross gill score, provided accurate measurements are possible. Multiple endpoints can provide improved understanding of the disease.

## Figures and Tables

**Table 1:** Examples of different challenge methods used in AGD experiments. C—cohabitation, DE—direct exposure to amoebae, GH—gill histology, GS—gill swabs, GGP—gross gill pathology, As—Atlantic salmon, NR—not reported.

Method	C—Ratio Infected:Naïve Fish, DE—Amoebae Concentration (L^−1^)	Aim	Endpoint (days)	Measurement of AGD Severity	Time to AGD Onset (AGD) or Morbidity (M) (days)	Temperature (°C)	Fish Species/Biomass (kg/m^3^)	Reference
C	15:40	Acquired resistance	84	GH	28 (M)	≥14	As/NR	[42]
C	20:60	Response to amoebae antigens	21	GH	21 (AGD)	≥14	As/NR	[28]
C	15:40	Glycoprotein Chemistry	7	GH	3 (AGD)	15–17	As, Brown trout Rainbow trout/30	[39]
C	15:40	Oral L-cysteine ethyl ester to treat AGD	7	GH, Mucus viscosity	3 (AGD)	15–17	As/NR	[46]
C	1:3	Response to AGD antigens	7	GH, blood and cardiac muscle	4–7 (AGD)	15–16	As/NR	[40]
C	4:40	Differences between detection levels of swabs	21	GS	21 (AGD)	11–13	As/NR	[41]
DE	230, 2307, 23,077	Initiate AGD using infective amoebae harvested from gills	7	GH	7 (termination of experiment)	NR	As/4.6	[44]
DE	2640	CpG oligodeoxynucleotides as immunostimulants	16	GH	16 (M)	17	As/NR	[26]
DE	2500	Gill pathology	8	GH	3 (AGD)	19.2	As/4.5	[47]
DE	10, 25, 50, 100, 500	Standardisation of laboratory-based infection model	14	GGP	14 (AGD)	16–16.5	As/NR	[45]
DE	3300	Innate immune response during AGD	11	GH	6 (AGD)	NR	As/NR	[27]
DE	1152	Potential of ɮ -glucans	72	GH	27 (M)	16–16.5	As/NR	[48]
DE	300	Impact of salmonid gill bacteria	8	GH	8 (AGD)	16–16.5	As/NR	[49]
DE	10,000/1000	Pathogenesis of AGD	2, 4, 7, 10, 16	GH	2 (AGD)	15–16	As/NR	[43]
DE	500	Seawater acclimation time	35	GH	23 (M)	16	As/NR	[50]
DE	300	Bithionol as a treatment	28	GGP	7 (AGD)	16.7	As/4.38	[51]
DE	7000	Effect of antibiotics on inoculating amoebae	3	GGP	3 (AGD)	16–18	As/3(?)	[52]
DE	100	Branchial mechanical injuries	2, 4, 8, 16, 24, 32	GH, GGP	4 (AGD)	15.4	As/NR	[53]
DE	500	Different batches of *N. perurans* and fish stocking densities	38	GH	23 (5 kg/m^3^ stocking density) 29 (1.7 kg/m^3^ stocking density)	15.5–16.5	As/5.0 or 1.7	[19]
DE	500	Characterising surface glycans or glycoproteins of *Neoparamoeba*	39		13 (AGD)	12–15	As/NR	[54]
DE	250 then 358	Hydrogen peroxide	21	GH	14 (AGD)	15	As/28	[23]
DE	5000	Hydrogen peroxide	15, 21, 23, 28, 30	GH, PCR	3 (AGD)	10	As/9.68	[55]
DE	1100	Sublethal freshwater exposure	10	qPCR	4 (AGD)	15–16	As/7.6 and 10.2	[21]
DE	150	Hydrogen peroxide at different temperatures	28 (17 °C), 42 (12 °C), 56 (8 °C)	GGP	NR	8, 12, 17	As/40 (during challenge), NR during trial	[56]
DE	1800	Gene expression	2, 7, 14, 21	GGP	<2 (AGD)	10.5–11.5	As/6.38	[57]
DE	100	Interaction between temperature and dose of hydrogen peroxide	15	GGP, PCR	11 (AGD)	16	As/22.11	[58]
DE	501	Novel diets	74	GH, PCR, Survival	NR	NR	As/8.2	[8]
DE	1200	Cyclic hypoxia	10	qPCR	2 (AGD)	18	As/NR	[59]
